# Single-cell RNA-Seq analysis of molecular changes during radiation-induced skin injury: the involvement of Nur77

**DOI:** 10.7150/thno.100417

**Published:** 2024-09-09

**Authors:** Tao Yan, Ping Yang, Hao Bai, Bin Song, Yulan Liu, Jiajia Wang, Yuehua Zhang, Wenling Tu, Daojiang Yu, Shuyu Zhang

**Affiliations:** 1 The Second Affiliated Hospital of Chengdu Medical College, Nuclear Industry 416 Hospital, Chengdu 610051, China.; 2 Laboratory of Radiation Medicine, West China School of Basic Medical Sciences & Forensic Medicine, Sichuan University, Chengdu 610041, China.; 3 NHC Key Laboratory of Nuclear Technology Medical Transformation (Mianyang Central Hospital), Mianyang 621099, China.; 4 Medical College of Tibet University, Lasa 850000, China.; 5 School of Bioscience and Technology, Chengdu Medical College, Chengdu 610500, China.; 6 Department of Burn and Plastic Surgery, Affiliated Hospital of Jiangnan University, Wuxi 214122, China.

**Keywords:** ionizing radiation, skin, radiation-induced skin injury, single-cell RNA sequencing (scRNA-Seq), orphan nuclear receptor 77 (Nur77)

## Abstract

**Introduction:** Ionizing radiation has been widely used in industry, medicine, military and agriculture. Radiation-induced skin injury is a significant concern in the context of radiotherapy and accidental exposure to radiation. The molecular changes at the single-cell level and intercellular communications during radiation-induced skin injury are not well understood.

**Methods:** This study aims to illustrate this information in a murine model and human skin samples from a radiation accident using single-cell RNA sequencing (scRNA-Seq). We further characterize the functional significance of key molecule, which may provide a potential therapeutic target. ScRNA-Seq was performed on skin samples from a nuclear accident patient and rats exposed to ionizing radiation. Bioinformatic tools were used to analyze the cellular heterogeneity and preferential mRNAs. Comparative analysis was performed to identify dysregulated pathways, regulators, and ligand-receptor interactions in fibroblasts. The function of key molecule was validated in skin cells and in three mouse models of radiation-induced skin injury.

**Results:** 11 clusters in human skin and 13 clusters of cells in rat skin were depicted respectively. Exposure to ionizing radiation caused changes in the cellular population (upregulation of fibroblasts and endothelial cells, downregulation of keratinocytes). Fibroblasts and keratinocytes possessed the most interaction pairs with other cell lineages. Among the five DEGs common to human and rat skins, *Nur77* was highly expressed in fibroblasts, which mediated radiosensitivity by cell apoptosis and modulated crosstalk between macrophages, keratinocytes and endothelial cells in radiation-induced skin injury. In animal models, *Nur77* knock-out mice (*Nur77*^-/-^) showed more severe injury after radiation exposure than wild-type counterparts in three models of radiation-induced skin injury with complex mechanisms.

**Conclusion:** The study reveals a single-cell transcriptional framework during radiation-induced skin injury, which provides a useful resource to uncover key events in its progression. *Nur77* is a novel target in radiation-induced skin injury, which provides a potential therapeutic strategy against this disease.

## Introduction

Ionizing radiation has been widely used in industry, medicine, science, military and agriculture, which has significantly increased the potential of uncontrolled exposure to radiation. The skin is the human body's largest organ, which serve as a physical barrier, protecting our bodies from potential assault by foreign organism or toxic substances 1. Skin has a strong ability to proliferate, which makes it more sensitive to radiation exposure 2, 3. Radiation-induced skin injury is a frequently fatal consequence of exposures from nuclear accidents 4. Moreover, radiotherapy is now a commonly employed technique for a variety of cancers 5. Radiation-induced skin injury, is a common and nearly inevitable complication in cancer patients undergoing radiotherapy. It is estimated that approximately 95% of patients receiving radiotherapy, develop moderate-to-severe skin reactions 6, 7.

The primary mechanisms underlying radiation-induced skin injury are linked to DNA damage, the excessive production of reactive oxygen species (ROS) 8, 9, metabolic alterations, protein turnover, cellular senescence, cell death, and vascular atrophy 10, 11. Unlike burns and scalds, ionizing radiation inflicts direct damage to both the skin and its deeper tissues, leading to inflammation, dryness, loss of elasticity, capillary dilation, pigmentation changes, and soft tissue fibrosis 12. Common clinical manifestations of radiation-induced skin injury include edema, erythema, dermatitis, dyspigmentation, ulcers, and fibrosis 6. Additionally, the irradiated skin of patients tends to have prolonged healing times and increased susceptibility to infections. Ultimately, these lesions can progress to fibrosis of the skin tissue and, in some cases, become cancerous, significantly impairing patients' quality of life. Although various topical, oral, and intravenous agents, along with physical therapies, are employed to prevent or treat radiation-induced skin injury, this condition continues to pose a significant clinical challenge 13.

Acute radiation-induced skin injury primarily involves cellular alterations and inflammation in the epidermis and the dermis. Ionizing radiation incites signaling between the epidermis and dermis through resident skin cells 14. Fibroblasts are the predominant cell type in dermis. Fibroblast heterogeneity is extensive between organs and inside an organ, which enables fibroblast diverse functions and roles in health maintenance. Fibroblasts are resident cells of many tissues and play a crucial role in wound healing and fibrosis 15. Fibroblast stimulation is involved in acute, late, and healing of radiation skin injury oxidative stress is generated at the time of radiation exposure, as well as days after irradiation due to propagation of free radicals and inflammatory cell recruitment, creates and antioxidant imbalance 8.

Our previous studies utilizing omics-based approaches have provided novel insights into radiation-induced injury, contributing to the development of effective countermeasures 7, 16-18. Traditional bulk RNA sequencing offers a representation of the collective gene expression of cells and tissues at a population level; however, this method is insufficient for elucidating the heterogeneity and plasticity of skin cells 19. Recent advances in single-cell RNA sequencing (scRNA-Seq) have enabled the analysis of transcriptomes from tens of thousands of cells at a single-cell resolution 20. In contrast to the average expression of genes derived from a mixed cell population obtained via bulk RNA-seq, large-scale scRNA-Seq allows for an unbiased assessment of cellular heterogeneity and regulatory networks at an unprecedented scale and resolution 21. The application of scRNA-Seq technology enables researchers to investigate gene expression patterns at the individual cell level, providing a more detailed characterization of the molecular profiles of study subjects, including cellular mapping and expression features 22.

Nur77, also known as NR4A1, TR3, or NGFI-B, is an orphan nuclear receptor encoded by the immediate early gene Nr4a1 23. It belongs to the steroid/thyroid/retinoid superfamily and plays a crucial role in cell proliferation, differentiation, and apoptosis 24. As a transcription factor, Nur77 regulates gene expression by binding to the promoters of its target genes. The members of the NR4A subgroup exhibit a high degree of conservation in the DNA binding domain (approximately 91%-95%) and the C-terminal ligand-binding domain (around 60%), while showing divergence in the N-terminal activation function (AF) region. These receptors can bind as monomers, homodimers, and heterodimers with retinoid X receptors (RXRs) to facilitate retinoid signaling, interacting with various permutations of the canonical nuclear receptor binding motif. Nur77 activates gene expression in a constitutive, ligand-independent manner 25. There is no report on the effect of Nur77 in radiation-induced skin injury.

In this study, we present the results of scRNA-Seq analysis of irradiated and non-irradiated skin samples from a patient and rats collected on different days post-irradiation. By utilizing well-defined cell lineage markers, we generated a comprehensive map that encompasses all cell types present in non-irradiated and irradiated skin tissues. A series of bioinformatic analyses were performed to identify dysregulated pathways, regulators, and ligand-receptor interactions within the skin tissues. Specifically, we characterized the role of Nur77 in radiation-induced skin injury. Our study provides insights into the progression of irradiated skin and identifies potential targets for medical therapies.

## Results

### Single-cell transcriptome profiling identified different skin cell types of rats

**Figure 1:**
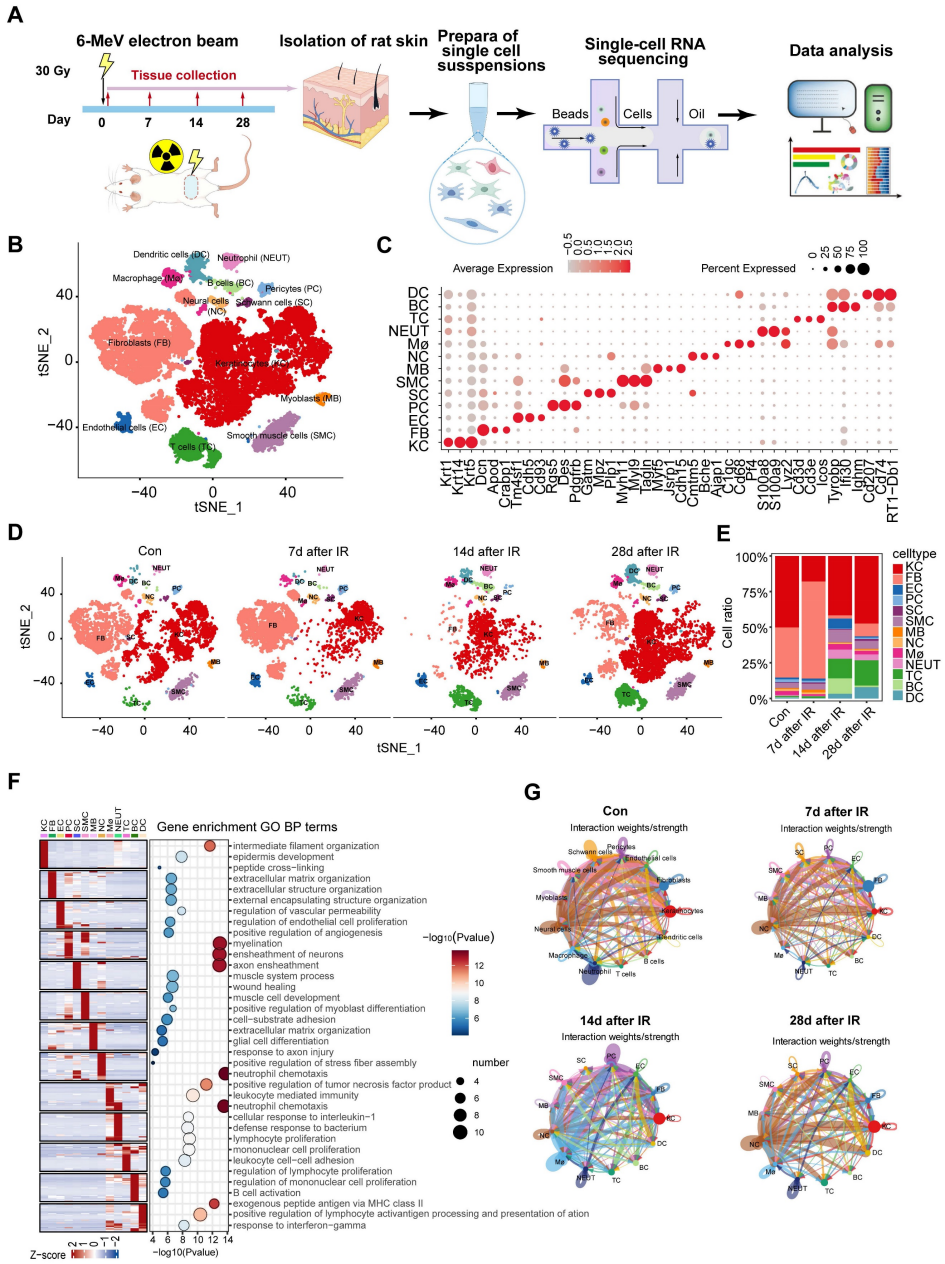
** Cell type identification by scRNA-Seq analysis in radiation-induced skin injury of rats.** (A) Schematic of the workflow showing the overall strategy of scRNA-Seq to create a rat mononuclear cell atlas and subjected to droplet-based 10x Genomics. (B) The t-SNE plot displays cell clusters with combined groups. Each dot represents a single cell. (C) Dot plot showing the expression of representative genes for each cell type. (D) The t-SNE plot displays main cell types in each group of skin tissues. (E) Bar plots show the proportions that each group contributes to each cluster. (F) Heatmap showing gene expression signatures of each cell type. (G) An overview of cell-cell interactions. Arrow and edge color indicate direction Circle network plots showing weights/strengths of cell-cell interactions generated with CellChat in different groups.

In the field of radiation therapy, patients may experience radiation-induced skin injuries that range from mild erythema to severe tissue necrosis 5. Despite extensive research into the causes of these injuries, effective management in clinical practice continues to pose a significant challenge, as an optimal treatment protocol has yet to be established 26. Furthermore, the changes in the skin that occur during the pathological progression of radiation-induced skin injury at various stages have not yet been thoroughly investigated. To probe the cellular and molecular dynamics of radiation-induced skin injury progression, we analyzed skin tissue from rat at day 0 (without radiation), day 7, day 14 and day 28 following a single dose of 30 Gy electron beam irradiation as reported previously 27, 28 (Figure 1A). This time points of sample collection consistent with the completion of wound re-epithelialization and the dose-response relationship in irradiated skin 29-31. The scRNA-Seq data from a total of 30,281 cells that met quality control standards were subjected to unsupervised clustering using t-distributed stochastic neighbor embedding (t-SNE) to elucidate cell-type composition at each time point (Figure S1A-B). Thirteen cell types were identified based on established markers specific to each type and their transcriptional characteristics (Figure 1B-1C, Figure S1C). The major cell types identified include: (i) Keratinocytes; (ii) Fibroblasts; (iii) Endothelial cells; (iv) Pericytes; (v) Schwann cells; (vi) Smooth muscle cells; (vii) Myoblasts; (viii) Neural cells; (ix) Macrophages; (x) Neutrophils; (xi) T cells; (xii) B cells; and (xiii) Dendritic cells. The key marker genes for each cell cluster are presented in Figure 1C. The predominant cell populations in the skin are comprised of keratinocytes and fibroblasts. The t-SNE plot in Figure 1D shows changes in different cell types at 7, 14, and 28 days as well as in unirradiated skin. There were significant differences in the proportions of some cell types among the different day of irradiation. In comparison to the control group, it was evident that 7 days post-radiation, fibroblast proportion was increased, while 14 days and 28 days post-radiation, fibroblast ratios decreased in rat skin tissues (Figure 1D-E, Figure S1D). Analysis of the top 30 marker genes for each cell type revealed unique transcriptional features and enriched pathways pertinent to their distinct physical functions (Figure 1F). For instance, Gene Ontology (GO) terms such as extracellular structure organization and ECM organization were found to be enriched in fibroblasts (Figure 1F). CellChat was utilized to analyze complex intercellular communications and to predict biologically relevant discoveries based on single-cell RNA sequencing data. Figure 1G and Supplementary Figure 1E presents the integrated cell-cell communication network, illustrating the intensity of interactions and the cell types that exhibit significant variations. Notably, fibroblasts and keratinocytes displayed the highest number of interaction pairs with cells from other lineages, underscoring their dominant roles in the irradiated rat skin tissues. Overall, our analyses elucidate the cellular landscape of various cell types during the progression of radiation-induced skin injury at single-cell resolution.

### A single-cell transcriptome atlas of irradiated human skin

**Figure 2:**
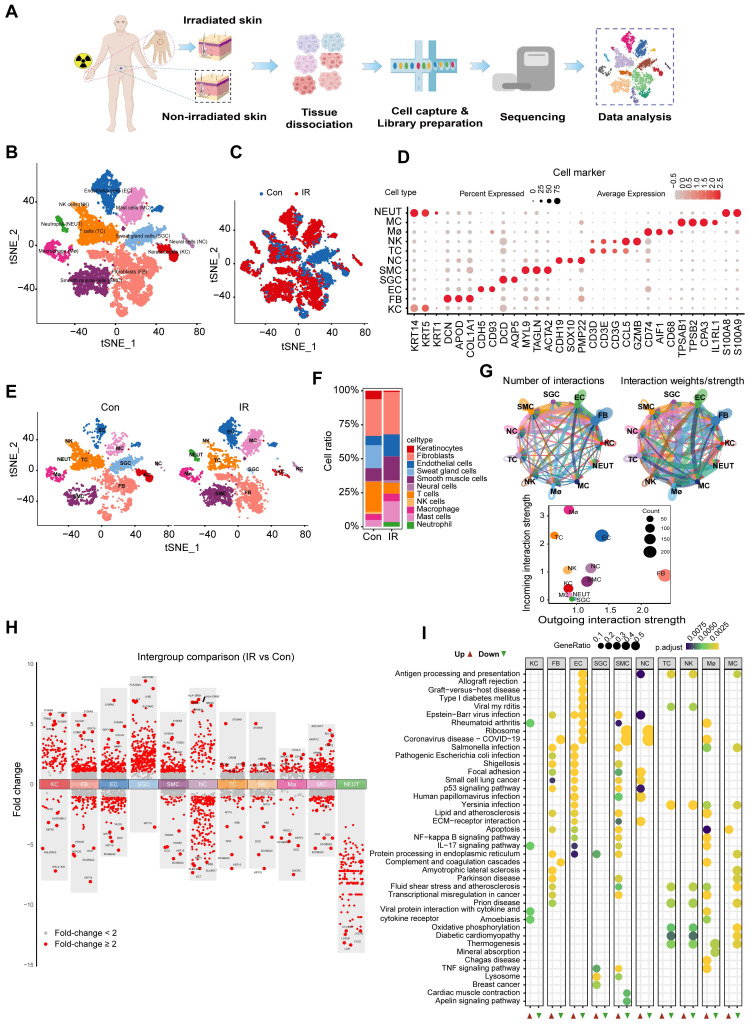
** Cutaneous cell type identification by scRNA-Seq analysis of a human patient in a radiation accident.** (A) Schematic of the workflow showing the overall strategy of scRNA-Seq to create a human mononuclear cell atlas and subjected to droplet-based 10x Genomics. (B) The t-SNE plot displays human skin cell clusters. Each dot represents only one cell. (C) The t-SNE plot displays human skin cell types with or without radiation. (D) Dot plot showing the expression of representative genes for each cell type. (E) The t-SNE plot displays human skins cell types with or without radiation. (F) Bar plots show the proportions that each group contributes to each cluster. (G) Circle network plots showing number (left) and weights/strengths of cell-cell interactions generated with CellChat. (H) Top five differential expressed genes (DEGs) with Fold change (FC) values. (I) KEGG (Kyoto Encyclopedia of Genes and Genomes) pathway enrichment analyses of DEGs (differentially expressed genes). Bubble plot of KEGG for up-regulated and down-regulated DEGs.

To confirm the uniformity of alterations in rat and human skin following irradiation, we conducted scRNA-Seq analysis on skin samples obtained from irradiated individuals (Figure 2A). A total of 17,002 sequenced skin cells met our quality control and inclusion criteria. Utilizing the 10× Genomics platform, we obtained approximately 8,647 and 8,355 transcriptomes for native and irradiated skin cells, respectively, resulting in a total of 56,144 detected genes. Unsupervised cluster analysis, performed using the Seurat software package, segregated the native and irradiated skin cells into 13 distinct clusters, which were overlaid and visualized in two-dimensional space using t-SNE (Figure 2B-C). The key marker genes for each cell cluster are presented in Figure 2D. Notably, there were significant differences in the proportions of certain cell types in skin tissue from patients with and without radiation exposure (Figure 2E-F). The results demonstrated the aggregated cell-cell communication network, indicating both the strength of interactions and the cell types exhibiting significant changes (Figure 2G). To identify radiation-induced alterations in gene expression for each cell type, we compared the differential expression profiles of control and irradiated groups. Additionally, we identified the top five genes with the highest fold change values (Figure 2H). To investigate the molecular pathways associated with radiation-induced skin injury, we performed Kyoto Encyclopedia of Genes and Genomes (KEGG) enrichment analyses on shared messenger RNAs (Figure 2I). Notably, fibroblasts exhibited the highest number of interaction pairs with cells from other lineages, underscoring their dominant roles in irradiated human skin tissues.

### Changes in the transcriptional profiles of fibroblast during radiation-induced skin injury

**Figure 3:**
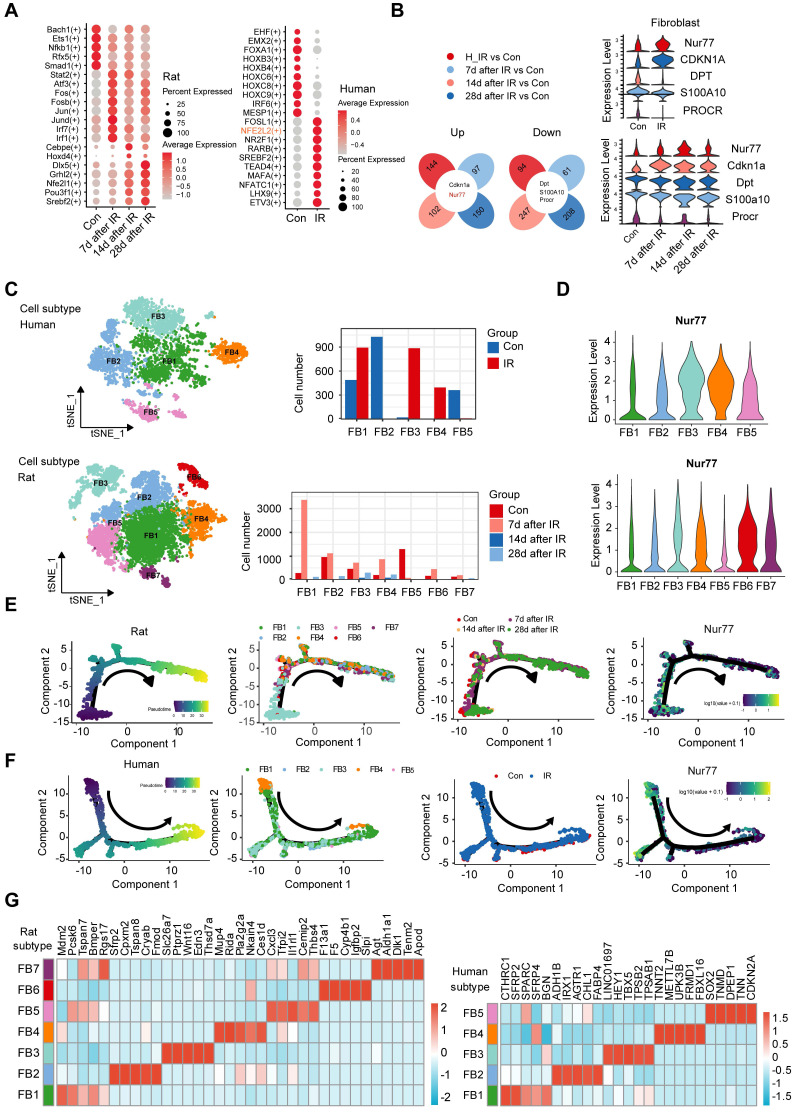
** Changes in the transcriptional profiles of skin fibroblast during radiation-induced skin injury.** (A) Bubble plot of TFs alterations in rat and human skin cells with or without radiation. (B) Venn plots showing the number of shared upregulated (upper) and downregulated (lower) DEGs between different groups of human and rat skin samples. Violin plots showing the expression levels of 5 common DEGs in human and rat skin. (C) The t-SNE plot displays rat (left) and human (right) fibroblast. Bar plots showing the cell number of each cell subtypes contributed. (D) Violin plot showing *Nur77* gene expression changes across different fibroblast subcluster in rat (left) and human (right). (E) Pseudotime ordering on rat fibroblasts and human fibroblasts (F) arranged them into a major trajectory, with two minor bifurcations. Each dot represents a single cell. The black arrow indicates the start and direction of the trajectory. Feature plots of expression distribution for Nur77 across pseudotime. (G) Heatmap showing the top 5 markers for fibroblast subcluster from the rat (left) and human (right) skin.

Transcription factors play vital roles in regulating gene expression under normal and stress conditions 32. To investigate the characterization of the transcription factors (TFs) involved under radiation conditions, we conducted an enrichment analysis of all TF from human and rat skin. This analysis revealed that 20 TF families were significantly enriched in this context (Figure 3A). We found that NFE2L2, FOSL1, NR2F1, RARB, ETV3, etc., were more enriched in irradiated skin cells than the healthy skin cells (Figure 3A). The transcription factor NFE2L2 is considered a master regulator of cellular homeostasis, as it controls the expression of numerous cytoprotective genes 33. After exposure to ionizing radiation, significant changes in radiation-induced skin injury were observed over time, particularly in fibroblasts. To further explore the mechanism of cutaneous radiation injury at the cellular level, we divided the dataset into 4 different groups and compared gene expression patterns of individual skin cell types between groups. To facilitate comparisons across species, we employed a reference mapping algorithm to assign each rat gene to its corresponding human gene. For this analysis, we identified thousands of differentially expressed genes in at least one skin cell type in response to radiation. The results of the differentially expressed genes (DEGs) analysis in human skin and rat skin revealed a total of 238 DEGs in human skin, comprising 144 upregulated and 94 downregulated genes. In rat skin, 158 DEGs were identified 7 days post-radiation (97 upregulated and 61 downregulated), 349 DEGs were found 14 days post-radiation (102 upregulated and 247 downregulated), and 358 DEGs were observed 28 days post-radiation (150 upregulated and 208 downregulated). Notably, only five common DEGs were identified between human and rat skin, with two being upregulated and three downregulated. Among these, Nur77 was found to be highly expressed in fibroblasts. The results of this analysis are illustrated in a Venn diagram (Figure 3B).

To elucidate the heterogeneity and dynamics of fibroblasts in response to radiation, we performed unsupervised clustering on rat and human skin fibroblasts separately, revealing additional heterogeneity with seven subclusters (FB1 to FB7) for rat fibroblasts and five subclusters (FB1 to FB5) for human fibroblasts (Figure 3C). Each subcluster displayed distinct marker gene profiles. In rat skin tissues, the expression of Nur77 increased following irradiation, peaking on the 14th day post-radiation. Figure 3D illustrates that the expression of Nur77 after irradiation is primarily associated with the FB6 subpopulation in rat skin fibroblasts. Similarly, in irradiated patient skin, Nur77 expression was mainly linked to FB3 and FB4 subpopulations. While t-SNE analysis revealed heterogeneity among irradiated fibroblasts, we also sought to determine whether they share common differentiation trajectories. The ordering of cells in pseudo-time predominantly arranged rat fibroblasts into a major trajectory, featuring two minor bifurcations. Fibroblasts from various subclusters were broadly distributed across the pseudo-time space, with FB3 cells primarily occupying the left half of the major trajectory, while the remaining cells included FB1, FB2, FB4, FB5, FB6, and FB7. In rats with radiation-induced skin injury, the predominant fibroblast subpopulation transitions from FB3 (*Mdm2^high^*,* Pcsk6^high^*,* Bmper^high^*) to FB1 (*Sfrp2^high^*,* Cpxm2^high^*,* Tspan8^high^*,* Cryab^high^*,* Fmod^high^*) and FB4 (*Mup4^high^, Rida^high^*,* Ces1d^high^*) (Figure 3E). In individuals with radiation-induced skin injury, the primary fibroblast subpopulation shifts from FB4 (*TNNT2^high^*,* METTL7B^ high^*,* UPK3B^ high^*,* FRMD1^ high^*,* FBXL16^ high^*) to FB1 (*CTHRC1^high^*,* SFRP2^high^*) and FB2 (*ADH1B^high^*,* IRX1^high^*,* AGTR1^high^*,* FABP4^high^*), as illustrated in Figure 3F.

### *Nur77* is involved in the irradiation process of skin cells

**Figure 4:**
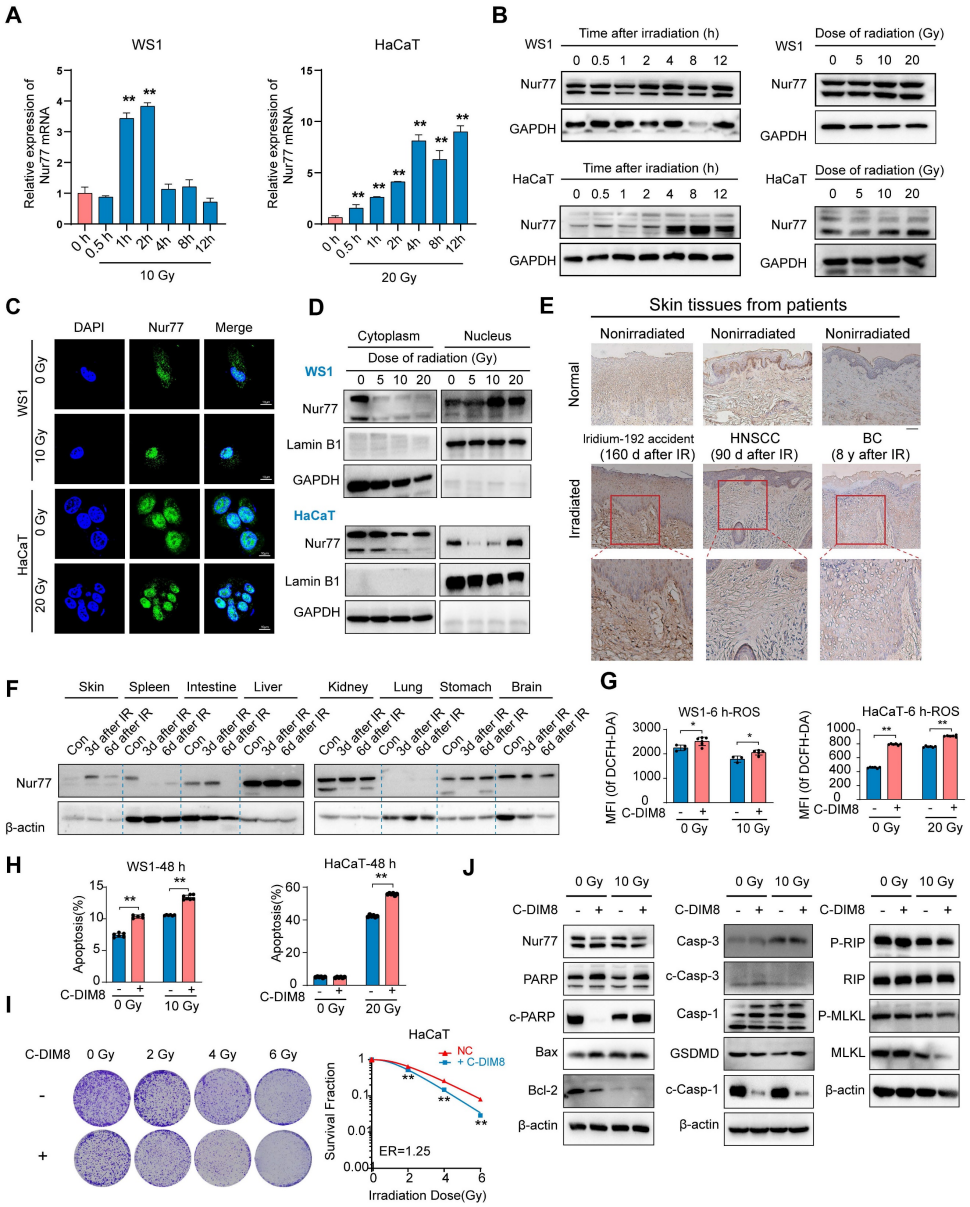
***Nur77* is involved in the irradiation process of skin cells.** (A) qRT-PCR analysis of *Nur77* mRNA expression in response to radiation in WS1 and HaCaT cells. (B) Western blotting analysis showing Nur77 expression in WS1 and HaCaT cells after different time post irradiation or different dose of irradiation. (C) and (D) Detection of radiation-induced nuclear-cytoplasmic distribution of Nur77 determined by preforming immunofluorescence analysis separating nuclear and cytoplasmic fraction and separating nuclear and cytoplasmic fractions. (E) Expression of Nur77 in normal and irradiated human skin tissues. (F) Western blot analysis showing the distribution of Nur77 in different organs and changes over time from 3 to 6 days after irradiation. (G) The effect of C-DIM8 on ROS production after different dose of irradiation as determined by DCFH-DA staining in WS1 and HaCaT cells. (H) The effect of C-DIM8 on cell apoptosis determined by AV/PI staining in WS1 and HaCaT cells. (I) The effect of Nur77 inhibitor C-DIM8 on radiosensitivity as determined by a colony formation assay following different doses of radiation. (J) Western blotting analysis showing cell death-related biomarker expression in irradiated WS1 cells treated with C-DIM8. *P < 0.05 and **P < 0.01, compared with the control group. Scale bar = 200 μm.

There is currently no research on the effect of Nur77 in radiation-induced skin injury. To investigate the biological effects of Nur77 in skin cells exposed to varying doses of ionizing radiation over different time intervals, we analyzed the mRNA and protein levels of Nur77 in human keratinocyte HaCaT cells and human skin fibroblast WS1 cells using western blotting and qRT-PCR, respectively (Figure 4A-B). The results indicated that exposure to 10 Gy X-rays radiation significantly upregulated Nur77 expression in WS1 cells, while 20 Gy radiation elicited a similar effect in HaCaT cells (Figure 4A). qRT-PCR analysis revealed that the mRNA level of Nur77 increased 1 hour after exposure to 10 Gy radiation, but decreased 4 h post-irradiation in WS1 cells. In HaCaT cells, the mRNA level of Nur77 exhibited a time-dependent increase; however, it decreased after 8 h of exposure to 20 Gy radiation (Figure 4A).

Additionally, western blotting analysis confirmed that Nur77 expression was time-dependent, with protein levels increasing at various time points following radiation exposure (Figure 4B). Subsequently, we investigated the localization of Nur77 post-irradiation using immunofluorescence staining. The results demonstrated that Nur77 is predominantly localized in the nucleus, with its expression levels increasing after irradiation (Figure 4C, Figure S1F). Furthermore, the protein level of Nur77 in the nuclear lysate was also markedly elevated (Figure 4D). In summary, ionizing radiation induces Nur77 expression and enhances its nuclear localization. To investigate the involvement of Nur77 in radiation-induced skin injury and fibrosis, we assessed the expression of Nur77 in the skin tissues of a human patient following irradiation, utilizing immunohistochemistry (IHC). The results indicated that Nur77 expression was significantly higher in the irradiated skin tissues compared to their nonirradiated counterparts, observed in both the epidermis and dermis (Figure 4E). Additionally, we examined the levels of Nur77 expression in various rat tissues at different time points post-radiation exposure. As illustrated in Figure 4F, Western blot analysis in a rat model revealed a significant upregulation of Nur77 protein levels in skin, hepatic, renal and gastric tissues following irradiation. In contrast, a notable reduction in Nur77 expression was observed in splenic, intestinal and cerebral tissues. To ascertain the pathway(s) involved in the degradation of Nur77. HaCaT and WS1 cells were incubated with proteosome inhibitor MG132 or autophagy inhibitor CQ, respectively. The results indicated that proteosome pathway and autophagy were possibly involved in Nur77 degradation (Figure S1G).

To characterize the role of Nur77 in cellular radiosensitivity, HaCaT and WS1 cells were pretreated with an Nur77 inhibitor (C-DIM8), and cell radiosensitivity was evaluated. 20 μM C-DIM8 was selected, because this dose did not induce significant toxicity to the cells. Pre-treatment with C-DIM8, followed by X-rays irradiation, significantly increased the levels of reactive oxygen species (ROS), both with and without radiation (Figure 4G, Figure S1H). Furthermore, the percentage of apoptosis in WS1 and HaCaT cells were markedly elevated in C-DIM8-treated cells (Figure 4H, Figure S1I). Additionally, we conducted a clonogenic survival assay to evaluate the effect of C-DIM8 on the radiosensitivity of HaCaT cells. The results showed that C-DIM8 pretreated exhibited higher radiosensitivity than control cells pretreated with DMSO alone (Figure 4I). Western blotting analysis showed that C-DIM8 promoted the expression of apoptosis related proteins (including PARP, cleaved PARP, C-caspase-1 and cleaved caspase3), but not necroptosis related proteins (Figure 4J). Taken together, these results suggested that Nur77 is involved in the irradiation process of skin cells.

### Loss of *Nur77* aggravates radiation-induced skin injury *in vivo*

**Figure 5:**
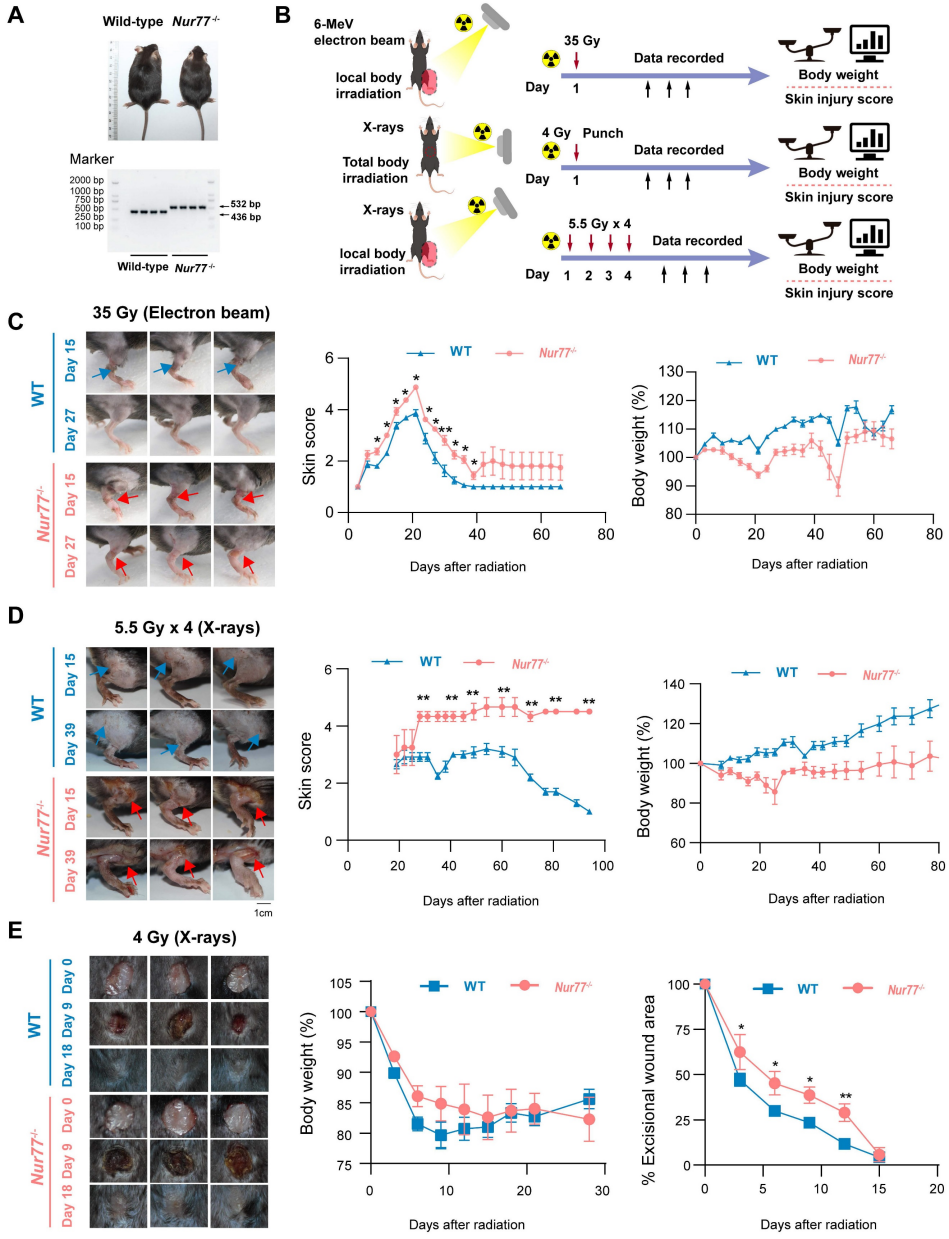
** Loss of *Nur77* aggravates radiation-induced skin injury in mouse models.** (A) The phenotypes and genotypes of wild-type (*Nur77*^+/+^) and *Nur77* knockout (*Nur77*^-/-^) mice. (B) Schematic of the workflow showing the establishment of the three mouse models with radiation-induced skin injury. 1) Acute radiation-induced skin injury; 2) Radiation fractionation; 3) A mouse model of full-thickness skin wounds combined with 4 Gy total-body irradiation. Loss of *Nur77* aggravates radiation-induced skin injury in three mouse models. (C) Pictures showing the weight change and the scoring curves of the whole course of radiogenic injury in mice with different *Nur77* genotypes. (D) Wound healing, body weight, and wound score results of radiation fractionation model in wild-type and *Nur77* knockout mice. (E) Wound healing, body weight, and wound healing score results of the radiation combined injury model in mice. *P < 0.05 and **P < 0.01, compared with the control group.

To confirm whether *Nur77* plays a role in skin reaction after radiation *in vivo*, wild-type and *Nur77* knock-out mice were used. Mice with *Nur77* ablation showed normal development and normal skin structure (Figure 5A). We established three mouse models of radiation-induced skin injury as reported previously (Figure 5B): (1) Radiation-induced acute skin injury by a single dose; (2) Radiation-induced acute skin injury by fractional doses; (3) A mouse model of full-thickness skin wounds combined with 4 Gy total-body irradiation 34-36. In the first model with a single dose of 35 Gy electron beam irradiation to generate radiogenic skin injury, which were further graded on a scale of 1 (no damage) to 5 (severe damage) as previously described 4. The onset of skin injury in mouse skin occurs 9 days following exposure to 35 Gy of irradiation, peaking at 30 days before the wounds commenced the healing process. The *Nur77* knock-out mice exhibited a heightened degree of radiation-induced skin injury compared to the wild-type mice (Figure 5C). Ulcers and dermatitis were noted in *Nur77* knock-out mice on day 39, whereas these conditions were not observed in the wild-type mice. In addition, to clarify the role of Nur77 ablation in accelerating tissue injury, another model exposed to fractionated radiation (5.5 Gy X-rays x 4 times) was established (Figure 5D). Similar to the results obtained with mice treated with 35 Gy electron beam irradiation. The removal of *Nur77* resulted in increased severity of skin damage caused by fractional X-rays exposure (Figure 5D). Concurrently, a combined injury model was developed through the administration of total body irradiation (TBI) at a dose of 4 Gy X-rays irradiation in conjunction with wounds on the dorsal region of mice. (Figure 5E). As expected, *Nur77* ablation aggravation wound healing in the combined injury mouse model (Figure 5E). These findings demonstrated that the absence of *Nur77* disrupts the development of radiation-induced skin injury and the subsequent healing mechanisms *in vivo*.

### *Nur77* affects the progression of radiation-induced skin injury through complex mechanisms

**Figure 6:**
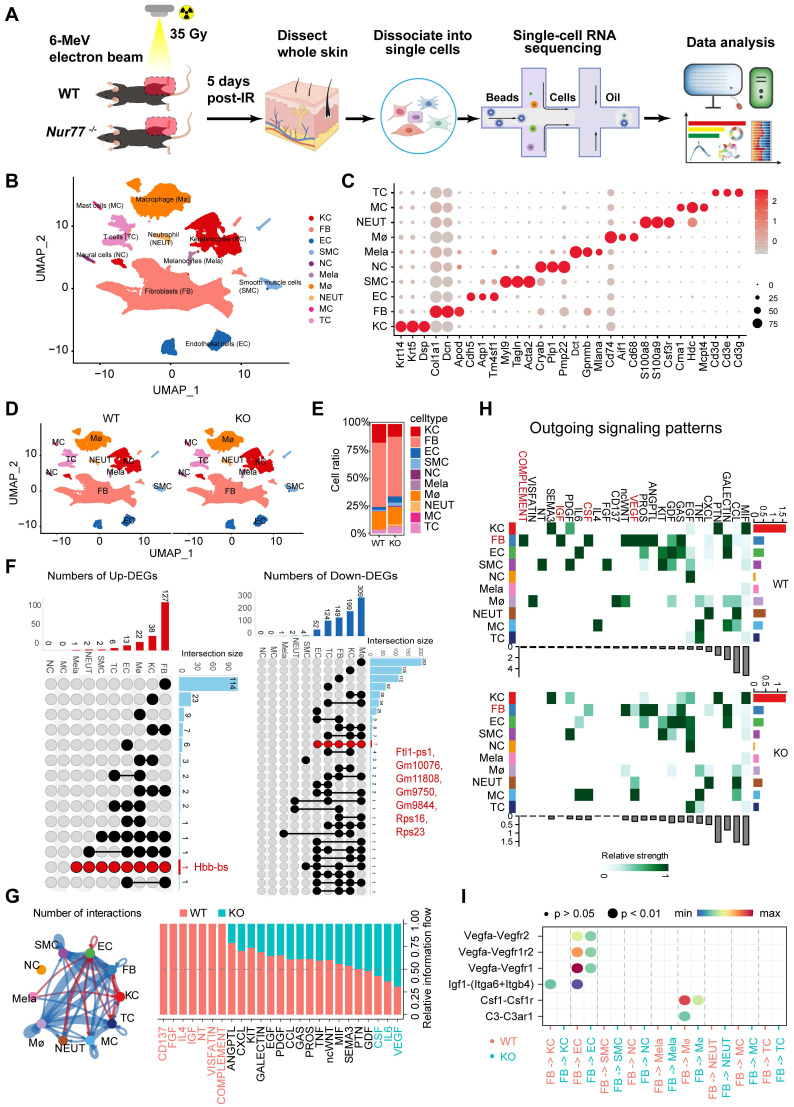
** scRNA-Seq reveals the complex mechanism by which *Nur77* mediates radiation-induced skin injury.** (A) Diagram displaying the process of sequencing single cells from radiation-induced skin injury samples obtained from wild-type (*Nur77*^+/+^) and *Nur77* knockout (*Nur77*^-/-^) mice. (B) The t-SNE plot displays main cell types in wild-type and *Nur77* knockout mice. Each dot represents only one cell. (C) Dot plot showing the expression of representative genes for each cell type. (D) The U-MAP plot displays cell types mouse skin with or without radiation. Each dot represents only one cell. (E) Bar plots show the proportions that each group contributes to each cluster. (F) The Venn diagram shows the number of up-regulated DEpcGs and down-regulated DEpcGs in different cell types. (G) Significant signaling pathways were ranked based on differences in the overall information flow within the inferred networks between *Nur77*^-/-^ and *Nur77*^+/+^ mouse skin. The overall information flow of a signaling network is calculated by summarizing all communication probabilities in that network. An overview of cell-cell interactions. Arrow and edge color indicate direction. Bar plots showing overall information flow of each signaling pathway. (H) Heatmap shows outgoing signaling patterns of *Nur77*^-/-^ and *Nur77*^+/+^ mouse skin. (I) Comparison of the significant ligand-receptor pairs between *Nur77*^-/-^ and *Nur77*^+/+^ mouse skin, which contribute to the signaling from fibroblast to other cells.

To elucidate the underlying mechanisms of *Nur77*'s effect, skin samples were collected from both non-irradiated and irradiated mice five days post-ionizing radiation (35 Gy) for scRNA-seq (Figure 6A). A total of 51,872 sequenced skin cells met our quality control and inclusion criteria. Utilizing classic markers for various cell types, we identified and visualized 10 clusters in two-dimensional space using UMAP (Figure 6B). The key marker genes for each cell cluster are illustrated in Figure 6C. Notably, significant differences exist in the proportions of specific cell types in Nur77 knockout mice, both following irradiation and in non-irradiated conditions, as depicted in Figures 6D-E. A total of 173 significantly upregulated differentially expressed genes (DEGs) and 840 downregulated DEGs were identified (Figure 6F). Figure 6G illustrates the aggregated cell-cell communication network, highlighting the interaction strength and the cell types exhibiting significant changes. Our investigations into cellular dynamics revealed a reduced interplay between fibroblasts and macrophages. Subsequently, we compared the information flow for each signaling pathway between Nur77-deficient and wild-type murine models (Figure 6G). The information flow for a given signaling pathway is defined as the sum of communication probabilities among all pairs of cell groups within the inferred network. We observed that certain pathways, including SEMA3, PTN, and GDF, maintain similar flow between the two groups. This suggests that these pathways are equally significant in the maturation of the skin in both groups. In contrast, other pathways exhibit significant changes in their information flow at *Nur77*^-/-^ compared to *Nur77*^+/+^: (1) pathways that are turned off (such as CD137, FGF, IL4, IGF, NT, VISFATIN, COMPLEMENT); (2) pathways that show a decrease (including ANGPTL, CXCL, KIT, GALECTIN, EGF, PDGF, CCL, GAS, PROS, TNF, ncWNT, MIF); and (3) pathways that experience an increase (such as CSF, IL6 and VEGF). To further elucidate these differential interactions, a heatmap analysis was conducted (Figure 6H). The heatmap revealed complex networks of cell-cell interactions between *Nur77*^-/-^ and *Nur77*^+/+^. Four pathways-VEGF, CSF, IGF, and COMPLEMENT-were found to be significantly inactive in *Nur77*^-/-^ mouse skin fibroblasts, suggesting that Nur77 may play a critical role in the progression of these pathways, as illustrated in Figure 6H. A comparison of significant ligand-receptor pairs between *Nur77*^-/-^ and *Nur77*^+/+^ mouse skin highlights their contributions to signaling from fibroblasts to other cell types. Specifically, the ligand lgf1 and its multi-subunit receptor ltga6/ltgb4 were found to act as major mediators of signaling between fibroblasts and keratinocytes (Figure 6I). Ligand Vegfa and its receptor Vegfr2/Vegfr1r2/Vegfr1, were also found to be low active in *Nur77*^-/-^ mouse skin for the signaling from fibroblast to Endothelial cells. Ligand Csf1 and its receptor Csf1r, ligand C3 and its receptor C3ar1, were found to be low active in *Nur77*^-/-^ mouse skin for the signaling from fibroblast to macrophage (Figure 6I). Previous research has demonstrated that fibroblasts can assist macrophages in maintaining homeostasis and responding to inflammatory conditions by supplying CSF1, which enhances macrophage survival, stimulates proliferation, or induces specific transcriptional profiles. Moreover, fibroblasts and macrophages can engage in indirect communication through interactions with the extracellular matrix (ECM), where fibroblasts are responsible for ECM deposition and organization, while macrophages efficiently degrade and modify it 37. CellChat prediction suggests *Nur77* may be involved in the pathogenesis of radiation-induced skin injury.

## Discussion

The skin is particularly susceptible to radiation because it is a constantly renewing organ, characterized by the rapid multiplication and maturation of cells 8. Consequently, radiation-induced skin injury poses a significant concern during both radiotherapy and radiation accidents 38. This type of injury differs from traditional wounds in that the healing process is complex and there is an elevated risk of recurrence 8. Although the skin's sensitivity to damage from radiation therapy and accidental exposure has been recognized for some time, the underlying mechanisms responsible for radiation-induced skin injury remain unclear, and the need for effective treatment solutions is still largely unresolved. Our previous studies have demonstrated that fatty acid metabolism play a role in radiation-induced skin damage 17. Traditional transcriptome research primarily acquires gene expression data through RNA sequencing (RNA-Seq) conducted on cell populations. In contrast, scRNA-Seq offers a more nuanced and detailed perspective, uncovering subtle differences among individual cells and elucidating complex biological processes. With the power of scRNA-seq, we were able to directly measure the transcriptome dynamics within different cell types following radiation exposure.

This study has identified previous unknown transcriptomic transitions, and cell communications in skin tissues from animal models and a human patient suffering from a radiation accident. Specifically, we observed that Nur77 is highly expressed in fibroblasts. In the context of radiation-induced skin damage, Nur77 plays a crucial role in regulating the radiosensitivity through mechanisms involving apoptosis and cross-talk among macrophages, keratinocytes, and endothelial cells. A recently study utilizing scRNA-Seq technology has indicated that aging-related IL-6 and IL-1 signaling in mouse skin, along with IL-17 upregulation and CCR6-mediated immune cell migration, play significant roles in irradiation-induced alopecia and dermatitis 39. However, this phenomenon has yet to be confirmed in human skin tissue due to the notable variances between mouse and human skin and hair. Murine and human skin share similarities in certain aspects; however, they also exhibit significant differences in key physiological processes, immune responses and wound repair mechanisms. Rodents have a distinctive panniculus carnosus layer-a thin muscle layer that is absent in humans, except for the platysma of the neck-which facilitates rapid wound contraction following injury 40. To avoid the bias of a single model, our study encompassed various species (mouse, rat and human skin tissues) to investigate transcriptional adaptations during radiogenic wound repair. However, the function and molecular mechanism of dysregulated genes, pathways and cell clusters remain poorly understood.

In the dysregulated mRNAs, we focused on multi-functional nuclear receptor *Nur77*. Ionizing radiation induces the expression of Nur77 and enhances its accumulation in the nucleus. Inhibition of Nur77 significantly elevates the production of ROS. Furthermore, Nur77 increases the radiosensitivity of skin cells by modulating apoptosis-related proteins. *In vitro* experiments demonstrate that *Nur77* plays a crucial role in cellular radiosensitivity. In subsequent experiments using three models of radiation-induced skin injury, *Nur77^-/-^* mice exhibited more severe damage following radiation exposure compared to *Nur77^+/+^* mice. These findings underscore the critical role of *Nur77* during radiation-induced skin injury, which provides a useful resource to uncover key events in its progression. In addition, we also verified the role of Nur77 in UV-induced skin damage. While UV radiation is fundamentally non-ionizing radiation, certain wavelengths overlap with X-rays, resulting in shared effects between UV and ionizing radiation 41. Results indicated that skin damage in *Nur77* deficient mice is more pronounced compared to wild-type mice (data not shown).

In recent years, the role of Nur77 in skin biology and related treatments has garnered increasing attention. Nur77 is crucial in skin wound healing and inflammatory responses 42. In the skin, Nur77 interacts with multiple signaling pathways, including the MAPK (mitogen-activated protein kinase) pathway and the STAT3 (signal transducer and activator of transcription 3) pathway. The MAPK signaling pathway is known to play an essential role in cell proliferation and differentiation, while STAT3 is associated with cell survival and proliferation 43. Nur77 influences skin cell behavior by regulating the activity of these pathways. The TGF-β signaling pathway is also vital in wound healing; however, studies indicate that Nur77 exhibits a negative regulatory role in this pathway, suggesting that it may inhibit the healing process under specific pathological conditions 44. This dual regulatory property renders Nur77 a complex yet valuable target. Further exploration of the specific mechanisms by which Nur77 interacts with different signaling pathways is essential to understand its effects on the migration and proliferation of skin cells, as well as its regulatory role in various pathological conditions. This understanding will lay the groundwork for the future development of novel therapies activating Nur77, potentially offering new hope for the treatment of various skin diseases.

In conclusion, this study provides a single-cell transcriptional framework during radiation-induced skin injury. Specifically, *Nur77* is a novel target in radiogenic skin injury, which provides a potential therapeutic strategy against this disease.

## Materials and methods

### Animals models

Protocols for experiments involving animals were approved by the Animal Experimentation Ethics Committee at Sichuan University (Sichuan, China). In all experiments or groups, animals were chosen randomly to undergo treatment. Male Sprague-Dawley (SD) rats (4 weeks old) were purchased from the Animal Center of Sichuan University (Sichuan, China). Rats were maintained in cages (23 °C) on a 12 h light/dark cycle with ad libitum access to food and water. All applicable international, national, and institutional guidelines for the care and use of animals were followed. Rats were anesthetized with an intraperitoneal injection of ketamine (75 mg/kg) and xylazine (10 mg/kg), and the hair on the rat buttock was shaved using a razor. A 3-cm-thick piece of lead was used to shield the rats and localize the radiation field (3 cm × 4 cm). A single 30 Gy dose 27, 28 of irradiation was administered to the treatment area at a rate of 1000 cGy/min using a 6-MeV electron beam accelerator (Varian VitalBeam™ linear accelerator; Varian Medical Systems, Inc., Palo Alto, GA). Nevertheless, this single dose radiation simulated the clinical skin lesions and the histological changes observed after fractionated irradiation 45, 46. The irradiated rat skin tissues were obtained at days 7, 14 and 28 post radiation, respectively, and another piece of skin was taken as control without radiation. The single cell RNA-Seq (scRNA-Seq) analysis were performed by OE Biotech (Shanghai, China), and to the sequencing Protocols for experiments involving mice were approved by the Animal Experimentation Ethics Committee at Sichuan University (Chengdu, China).

Male* Nur77* knock-out and wild-type controls on a C57BL/6 background were acquired from Cyagen (Suzhou, China). Male mice with an intraperitoneal injection of pentobarbital sodium (1%, 30 mg/kg), and the hair on hind limb of the mice was shaved using a razor and then immobilized with adhesive tape on a plastic plate to minimize motion during radiation exposure. We established three models with radiation-induced skin injury: 1) Acute radiation-induced skin injury; 2) Radiation fractionation; 3) A mouse model of full-thickness skin wounds combined with 4 Gy total-body irradiation. *Nur77* KO and wild-type mice received a single 35 Gy dose of irradiation was administered to the right hind leg at a rate of 1000 cGy/min using a 6-MeV electron beam accelerator (Varian VitalBeam™ linear accelerator; Varian Medical Systems, Inc., Palo Alto, GA). A 1-cm-thick piece of lead was used to shield the animals (*Nur77* KO and wild-type mice) and localize the radiation field on hind leg 5.5 Gy for continuous 4 days X-rays irradiation (X-rays 5.5 Gy × 4) using X-rays irradiator (320 KV; KUB Technologies, Inc., Stratford, CT) (n=6). *Nur77* KO and wild-type mice received 4 Gy total-body irradiation at a dose rate of 100 cGy/min using X-rays accelerator (320 KV; KUB Technologies, Inc., Stratford, CT). After irradiation, mice were anesthetized prior to wounding. A full-thickness skin wound with a diameter of 1 cm was created with a stainless-steel punch that was cleaned with 75% alcohol before each use. The skin injury recovery process in *Nur77* KO and wild-type mice were evaluated for 15 d, and pictures were taken of the wound site. The percentage of wound area was defined by the ratio of the residual wound area to that of the original wound. Skin reactions were followed at regular intervals using the semi-quantitative skin injury scale from 1 (no damage) to 5 (severe damage), as previously described 4.

### Tissue dissociation and cell isolation

Skin samples were collected and stored in ice-cold phosphate buffer saline (PBS, Sigma-Aldrich) after upper blepharoplasty. For cell isolation, subcutaneous tissues were removed from the samples. After a wash with cold PBS, samples were minced with scissors into ∼1 mm^2^ piece in PBS on ice and transferred into 15 mL centrifuge tubes, rinsed twice with cold PBS and incubated at 37 °C for 1 hr in digestion solution (PBS supplemented with 2 mg/mL collagenase I, 2 mg/mL collagenase IV, 2 mg/mL dispase and 0.125% trypsin-EDTA). Single-cell suspension was obtained by pipetting, and the suspension was passed through a 40 μm strainer (BD Falcon). The digestion was stopped by adding DMEM (Gibco) containing 10% fetal bovine serum (FBS, Gibco). Dissociated cells were collected by centrifugation at 300 g for 5 min at 4 °C and resuspended in 5 mL cold PBS. The cells were washed twice as above and resuspended in cold PBS supplemented with 10% FBS. The dissociated cells were then sorted by FACS (BD Influx) to deplete cell debris. Propidium iodide staining was used to exclude dead cells from the suspension of single cells. The generated single-cell suspensions in 50 μL 0.04% bovine serum albumin (BSA, Gibco) in PBS were used for 10x Genomic sequencing.

### Human skin samples

Human skin samples were obtained from a victim of an iridium radiation accident as reported previously 47. The skin samples were obtained 160 d after irradiation from the right hand, which was exposed to iridium-192 (192Ir) metal chain (with an activity of 966.4 GBq or 26.1 Ci). Normal skin tissues were obtained from the patient's navel. An irradiated head sample was obtained from the scalp of a 90 year old male patient with recurring squamous cell carcinoma (well differentiated) who was administered standard radiotherapy 90 d before the study. An irradiated breast skin sample was obtained from the margin of an ulcer in the tumor bed of a 67 year old female patient with breast cancer who had suffered endless pain for 8 years. The normal skin tissue counterparts were obtained when skin grafts from the dorsal myocutaneous flap were obtained during surgery. Informed consent for sample collection was obtained from the patient. Patients provided informed consent for this study. All patients provided written informed consent for their tissues to be used for scientific research. Ethical approval of the study was obtained from the China National Nuclear Corporation 416 Hospital (Chengdu, China).

### Cell cultures and irradiation

The HaCaT cell line (human keratinocytes) was obtained from the German Cancer Research Center (Heidelberg, Germany) as reported previously 7. The WS1 cell line (human skin fibroblast) was purchased from ATCC. Cells were maintained in Dulbecco's modified Eagle's medium (DMEM). All culture media were supplemented with 10% fetal bovine serum (FBS; Biological Industries, Kibbutz Beit-Haemek, Israel). The cells were grown at 37 °C in incubators with 5% CO_2_ and exposed to different dosages of ionizing radiation from an X-rays linear accelerator (KUBTEC XCELL 320, Milford, CT) at a fixed dose rate of 1.7 Gy/min, as previously reported 7, 17.

### RNA extraction and real-time PCR analysis

Total RNA was extracted from cells and tissues with Trizol reagent (Invitrogen, Carlsbad, CA). Total RNA from HaCaT and WS1 cells was reverse transcribed to cDNA using an oligo (dT) primer and Superscript II reverse transcriptase (Invitrogen). The SYBR green dye One Step TB Green® PrimeScript™ RT-PCR Kit (Takara, Toyobo, Japan) was used for amplification of cDNA. mRNA levels as well as that of the internal standard, *glyceraldehyde 3-phosphate dehydrogenase* (*GAPDH*), were measured by real-time quantitative PCR in triplicates using a Prism 7900 real-time PCR machine (Applied Biosystems, Foster City, CA). Real-time PCR analysis of the mRNAs was designed and conducted at AcebioX Biotech (Shanghai, China). The quantitative real-time PCR results were analyzed and expressed as relative mRNA levels based on the cycle threshold value, which was then converted to fold change 48. The primers used are listed in Table S1.

### Western blotting analysis

Cells were harvested in lysis buffer (BioTeke, Beijing, China) supplemented with 1 mM phenylmethylsulfonyl fluoride and 1 mM protease Inhibitor Cocktail for 20 min. After centrifugation at 4 °C for 10 min (12000 rpm), the supernatant was collected, and protein concentrations were measured using the BCA protein assay kit (Beyotime, Nantong, China). 30 μg protein was fractionated by 15% SDS-PAGE and electrophoretically transferred to polyvinylidene difluoride (PVDF) membranes (Millipore, Bedford, MA). After blocking with 5% non-fat milk in phosphate-buffered saline (PBS) containing 0.1% Tween-20 (PBST) for 1 h at room temperature, the membranes were blotted with primary antibodies against Nur77 (#3960, Cell Signaling Technology). β-actin (#ab8226, Abcam) and GAPDH (#60004-1-lg, Proteintech) were used as a loading control. After washing 4 times with PBST, the membrane was incubated with a horseradish peroxidase (HRP)-conjugated anti-rabbit (#A21020, Abbkine) or anti-mouse secondary antibody (#A21010, Abbkine) for 2 h. Protein bands were visualized and photographed using a FluroChem M imaging system (Shenhua, Hangzhou, China). Densitometry was performed using ImageJ v1.8.0-172 software (National Institutes of Health). The specific information of other antibodies was list in Table S2.

### Immunofluorescence assay

HaCaT and WS1 cells were fixed with 4% paraformaldehyde, washed with phosphate buffered saline, and permeabilized with 1% Triton X-100 in phosphate buffered saline. Cells were blocked with blocking buffer (phosphate buffered saline, 1% Triton X-100, and 5% BSA) and incubated at 4 °C with Nur77 (#NBP2-66980, Novus) antibody (1: 200) overnight, FITC-conjugated goat anti-mouse antibody (1:300) was added for 2 h at room temperature, and cells were dyed with 200 nM MitoTracker Red CMXRos (#C1035, Beyotime) at 37 °C for 30 min in darkness. Nuclei were counterstained with DAPI (#P0131, Beyotime).

### Radiosensitivity detection experiment based on clonogenic survival assay

Clonogenic survival assay of intestinal cells was performed as reported previously 49. HaCaT cells were seeded onto 6-well plates with different densities (500-4,000 cells per well) depending on the dose of irradiation, HaCaT cells were pretreated with C-DIM8 and then exposed to 0, 2, 4, and 6 Gy X-rays irradiation. After irradiation, the cells were grown for 7-10 d to allow for colony formation and were subsequently fixed and stained using crystal violet. Colonies consisting of 50 or more cells were counted as a clone. Colonies were observed using a microscope. Colony size was calculated using Image J, the surviving fraction (SF) was calculated as reported previously 50.

### Immunohistochemistry (IHC)

For the IHC analysis, the irradiated skin samples were obtained from the right leg of a patient who had been exposed to an iridium-192 (^192^Ir) irradiated metal chain (with an activity of 966.4 GBq or 26.1 Ci) 160 d before the study as reported previously 51. An irradiated head sample was obtained from the scalp of a 90 year old male patient with recurring squamous cell carcinoma (well differentiated) and administered standard radiotherapy 90 d before the study. An irradiated breast skin sample was obtained from the margin of an ulcer in the tumor bed of a 67 year old female breast cancer patient who had suffered endless pain for 8 years. The normal skin tissue counterparts were obtained when skin grafts from the dorsal myocutaneous flap were obtained during surgery. In addition, the irradiated skin sample used in the scRNA-Seq analysis was obtained from the hands of a patient who was accidentally exposed to X-rays irradiation approximately 250 d post irradiation (the dose was difficult to determine) showed obvious skin injury 3 d after irradiation. The irradiated skin sample was taken from the margin of the ulcer in the hand, and the normal skin tissue counterpart was obtained from a skin graft from the dorsal abdominal flap during surgery.

Protocols for experiments involving mice were approved by the Animal Experimentation Ethics Committee at Soochow University (Suzhou, China) and West China Second University Hospital (Chengdu, China). Human skin tissues were collected at the Nuclear Industry 416 Hospital. Informed consent for sample collection was obtained from the patients. Ethics approval for the study was obtained from Nuclear Industry 416 Hospital (Chengdu, China).

Other methods were detailed in the Supplementary Materials and Methods.

### Data availability

The scRNA-Seq data reported in this article have been deposited in the National Center for Biotechnology Information (NCBI) Gene Expression Omnibus (GEO) and are accessible through GEO Series accession number GSE193564 and GSE193807.

## Supplementary Material

Supplementary methods, figure and tables.

## References

[1] Kabashima K, Honda T, Ginhoux F, Egawa G (2019). The immunological anatomy of the skin. Nat Rev Immunol.

[2] Wang K, Tepper JE (2021). Radiation therapy-associated toxicity: etiology, management, and prevention. CA Cancer J Clin.

[3] Bray FN, Simmons BJ, Wolfson AH, Nouri K (2016). Acute and chronic cutaneous reactions to ionizing radiation therapy. Dermatol Ther (Heidelb).

[4] Brand RM, Epperly MW, Stottlemyer JM, Skoda EM, Gao X, Li S (2017). A topical mitochondria-targeted redox-cycling nitroxide mitigates oxidative stress-induced skin damage. J Invest Dermatol.

[5] Hegedus F, Mathew LM, Schwartz RA (2017). Radiation dermatitis: an overview. Int J Dermatol.

[6] Rosenthal A, Israilevich R, Moy R (2019). Management of acute radiation dermatitis: a review of the literature and proposal for treatment algorithm. J Am Acad Dermatol.

[7] Qiu Y, Gao Y, Yu D, Zhong L, Cai W, Ji J (2020). Genome-wide analysis reveals zinc transporter ZIP9 regulated by DNA methylation promotes radiation-induced skin fibrosis via the TGF-β signaling pathway. J Invest Dermatol.

[8] Ryan JL (2012). Ionizing radiation: the good, the bad, and the ugly. J Invest Dermatol.

[9] Hao Y, Li H, Guo J, Wang D, Zhang J, Liu J (2023). Bio-inspired antioxidant heparin-mimetic peptide hydrogel for radiation-induced skin injury repair. Adv Healthc Mater.

[10] Yu D, Cai W, An L, Feng Y, Cao J, Zhang S (2020). The application of a modified random flap in breast cancer patients after surgery and radiation. Asian J Surg.

[11] Kamat JP, Devasagayam TP (2000). Oxidative damage to mitochondria in normal and cancer tissues, and its modulation. Toxicology.

[12] Ashack KA, Kuritza V, Visconti MJ, Ashack L (2020). Dermatologic sequelae associated with radiation therapy. Am J Clin Dermatol.

[13] Feight D, Baney T, Bruce S, McQuestion M (2011). Putting evidence into practice. Clin J Oncol Nurs.

[14] Müller K, Meineke V (2011). Radiation-induced mast cell mediators differentially modulate chemokine release from dermal fibroblasts. J Dermatol Sci.

[15] Thulabandu V, Chen D, Atit RP (2018). Dermal fibroblast in cutaneous development and healing. Wiley Interdiscip Rev Dev Biol.

[16] Wang W, Luo J, Sheng W, Xue J, Li M, Ji J (2016). Proteomic profiling of radiation-induced skin fibrosis in rats: targeting the ubiquitin-proteasome system. Int J Radiat Oncol Biol Phys.

[17] Tu W, Tang S, Yan T, Feng Y, Mo W, Song B (2022). Integrative multi-omic analysis of radiation-induced skin injury reveals the alteration of fatty acid metabolism in early response of ionizing radiation. J Dermatol Sci.

[18] Xiao Y, Mo W, Jia H, Yu D, Qiu Y, Jiao Y (2020). Ionizing radiation induces cutaneous lipid remolding and skin adipocytes confer protection against radiation-induced skin injury. J Dermatol Sci.

[19] Stuart T, Satija R (2019). Integrative single-cell analysis. Nat Rev Genet.

[20] Zheng GX, Terry JM, Belgrader P, Ryvkin P, Bent ZW, Wilson R (2017). Massively parallel digital transcriptional profiling of single cells. Nat Commun.

[21] Kulkarni A, Anderson AG, Merullo DP, Konopka G (2019). Beyond bulk: a review of single cell transcriptomics methodologies and applications. Curr Opin Biotechnol.

[22] Wang S, Zheng Y, Li Q, He X, Ren R, Zhang W (2021). Deciphering primate retinal aging at single-cell resolution. Protein Cell.

[23] Hazel TG, Nathans D, Lau LF (1988). A gene inducible by serum growth factors encodes a member of the steroid and thyroid hormone receptor superfamily. Proc Natl Acad Sci U S A.

[24] Zhang XK (2007). Targeting Nur77 translocation. Expert Opin Ther Targets.

[25] Guo H, Golczer G, Wittner BS, Langenbucher A, Zachariah M, Dubash TD (2021). NR4A1 regulates expression of immediate early genes, suppressing replication stress in cancer. Mol Cell.

[26] Yang X, Ren H, Guo X, Hu C, Fu J (2020). Radiation-induced skin injury: pathogenesis, treatment, and management. Aging (Albany NY).

[27] Xue J, Yu C, Sheng W, Zhu W, Luo J, Zhang Q (2017). The Nrf2/GCH1/BH4 axis ameliorates radiation-induced skin injury by modulating the ROS cascade. J Invest Dermatol.

[28] Cao J, Zhu W, Yu D, Pan L, Zhong L, Xiao Y (2019). The involvement of SDF-1α/CXCR4 axis in radiation-induced acute injury and fibrosis of skin. Radiat Res.

[29] Plikus MV, Guerrero-Juarez CF, Ito M, Li YR, Dedhia PH, Zheng Y (2017). Regeneration of fat cells from myofibroblasts during wound healing. Science.

[30] Brown JM, Goffinet DR, Cleaver JE, Kallman RF (1971). Preferential radiosensitization of mouse sarcoma relative to normal skin by chronic intra-arterial infusion of halogenated pyrimidine analogs. J Natl Cancer Inst.

[31] Nolan E, Bridgeman VL, Ombrato L, Karoutas A, Rabas N, Sewnath CAN (2022). Radiation exposure elicits a neutrophil-driven response in healthy lung tissue that enhances metastatic colonization. Nat Cancer.

[32] Lambert SA, Jolma A, Campitelli LF, Das PK, Yin Y, Albu M (2018). The human transcription factors. Cell.

[33] Hayes JD, Dinkova-Kostova AT (2014). The Nrf2 regulatory network provides an interface between redox and intermediary metabolism. Trends Biochem Sci.

[34] Nawroth I, Alsner J, Behlke MA, Besenbacher F, Overgaard J, Howard KA, Kjems J (2010). Intraperitoneal administration of chitosan/DsiRNA nanoparticles targeting TNFα prevents radiation-induced fibrosis. Radiother Oncol.

[35] Cao J, Zhong L, Feng Y, Qian K, Xiao Y, Wang G (2021). Activated beta-catenin signaling ameliorates radiation-induced skin injury by suppressing marvel D3 expression. Radiat Res.

[36] Geng F, Chen J, Song B, Tang Z, Li X, Zhang S (2024). Chaperone- and PTM-mediated activation of IRF1 tames radiation-induced cell death and the inflammatory response. Cell Mol Immunol.

[37] Zhou X, Franklin RA, Adler M, Jacox JB, Bailis W, Shyer JA (2018). Circuit design features of a stable two-cell system. Cell.

[38] DiCarlo AL, Bandremer AC, Hollingsworth BA, Kasim S, Laniyonu A, Todd NF (2020). Cutaneous radiation injuries: models, assessment and treatments. Radiat Res.

[39] Paldor M, Levkovitch-Siany O, Eidelshtein D, Adar R, Enk CD, Marmary Y (2022). Single-cell transcriptomics reveals a senescence-associated IL-6/CCR6 axis driving radiodermatitis. EMBO Mol Med.

[40] Lio DCS, Chia RN, Kwek MSY, Wiraja C, Madden LE, Chang H (2020). Temporal pressure enhanced topical drug delivery through micropore formation. Sci Adv.

[41] Tang X, Yang T, Yu D, Xiong H, Zhang S (2024). Current insights and future perspectives of ultraviolet radiation (UV) exposure: Friends and foes to the skin and beyond the skin. Environ Int.

[42] Niu G, Ye T, Qin L, Bourbon PM, Chang C, Zhao S (2015). Orphan nuclear receptor TR3/Nur77 improves wound healing by upregulating the expression of integrin β4. Faseb j.

[43] Xie P, Yan LJ, Zhou HL, Cao HH, Zheng YR, Lu ZB (2022). Emodin protects against lipopolysaccharide-induced acute lung injury via the JNK/Nur77/c-Jun signaling pathway. Front Pharmacol.

[44] Banno A, Lakshmi SP, Reddy AT, Kim SC, Reddy RC (2019). Key functions and therapeutic prospects of Nur77 in inflammation related lcung diseases. Am J Pathol.

[45] Archambeau JO, Pezner R, Wasserman T (1995). Pathophysiology of irradiated skin and breast. Int J Radiat Oncol Biol Phys.

[46] Holler V, Buard V, Gaugler MH, Guipaud O, Baudelin C, Sache A (2009). Pravastatin limits radiation-induced vascular dysfunction in the skin. J Invest Dermatol.

[47] Song J, Zhang H, Wang Z, Xu W, Zhong L, Cao J (2018). The role of FABP5 in radiation-induced human skin fibrosis. Radiat Res.

[48] Zhang S, Hao J, Xie F, Hu X, Liu C, Tong J (2011). Downregulation of miR-132 by promoter methylation contributes to pancreatic cancer development. Carcinogenesis.

[49] Xie LW, Cai S, Zhao TS, Li M, Tian Y (2020). Green tea derivative (-)-epigallocatechin-3-gallate (EGCG) confers protection against ionizing radiation-induced intestinal epithelial cell death both in vitro and in vivo. Free Radic Biol Med.

[50] Xue J, Zhu W, Song J, Jiao Y, Luo J, Yu C (2018). Activation of PPARα by clofibrate sensitizes pancreatic cancer cells to radiation through the Wnt/β-catenin pathway. Oncogene.

[51] Yu D, Zhang S, Mo W, Jiang Z, Wang M, An L (2021). Transplantation of the stromal vascular fraction (SVF) mitigates severe radiation-induced skin injury. Radiat Res.

