# Supplementary material for: Single-cell RNA-Seq analysis of molecular changes during radiation-induced skin injury: the involvement of Nur77
Source: Theranostics. 2024 Sep 9;14(15):5809–25. doi: 10.7150/thno.100417 (PMC11426238; doi:10.7150/thno.100417)
Supplement: Supplementary file 1 — Supplementary methods, figure and tables. [file thnov14p5809s1.pdf]

Supplementary Methods

Processing raw data from scRNA-Seq of 10x Genomics

We used the default parameters of Cell Ranger single-cell software suite (v2.2.0) (10x Genomics) (https://support.10xgenomics.com/single-cell-vdj/software/pipelines/latest/system-requirements#header). The quality of the sample-specific FASTQ file was evaluated by the counts of Cell Ranger which were aligned to the human reference genome (hg19) using a STAR aligner to generate the digital gene expression matrix [1]. The expression level for each transcript was determined using the number of unique molecular identifiers (UMI) assigned to the transcript. The filtered gene expression matrices were then used for downstream analyses.

Bioinformatic analysis

Raw expression matrices were calculated by CellRanger toolkit (version.5.0 10× Genomics, Pleasanton, CA, USA) via alignment to human genome reference build GRCh38 (Ensembl 88). The low-quality cells were discarded according to following criteria: (1) cells had unique molecular identifier (UMI) less than 800 or more than 20,000; (2) cells had no more than 500 expressed genes; and (3) the mitochondrial genes should account for less than 15% UMI. Subsequently, the batch effect of donors was removed by applying integration workflow wrapped in Seurat (version 4.0, New York, NY, USA). Briefly, we first constructed a reference with finding “anchors” among batches/individuals by reciprocal PCA reduction. Then, we split the combined object by each donor and performed log normalization prior to finding anchors. The UMI count was normalized by the “NormalizeData” function. The top 3000 highly variable genes (HVGs) were calculated with “FindVariableFeatures” and

selected for downstream analysis. Data were scaled with the "ScaleData" function, setting the parameter "vars.to.regress" to "percent.mito" and "nUMI". Principal component analysis (PCA) was performed using the "RunPCA" function with the top 3000 HVGs. To cluster single cells into subsets, we adopted unsupervised graph-based clustering algorithm implemented in Seurat package. "PCElbowPlot" function was used to choose the number of PCs and a shared nearest-neighbor (SNN) graph was constructed using the "FindNeighbors" function with the top 35 PCs. Lastly, cells were clustered by the "FindClusters" and "RunUMAP" functions. "FindAllMarkers" function was applied to detect signature genes for each cluster with setting the parameter "min.pct" to 0.3 and "logfc.threshold" to 0.4. Subsequently, cell clusters were annotated manually to the major cell types according to known markers. Any cluster with multiple markers of two types of cells was manually discarded as a doublet.

scRNA-Seq data analysis and cell-type identification

Seurat R package (version 2.3.4) [2] was used to further analyze the single-cell RNA-seq data. After the initial Cell Ranger metric assessment, cells with fewer than 500 genes or more than 5,000 genes detected, and more than 10% mitochondrial genes were further excluded from the downstream analyses. After quality control, 35,678 cells remained and were used for downstream bioinformatic analyses. Sequencing reads for each gene were normalized to total UMIs in each cell to obtain normalized UMI values by "NormalizeData" function. In other words, the UMI counts of each gene was then divided by the total UMIs of the cell and multiplied by the median of the total UMI, and transformed by the natural logarithm. The "ScaleData" function was used to scale and center expression levels in the

data set for dimensional reduction. To avoid batch effects among samples and experiments, the top 1,100 highly variable genes were used for canonical correlation analysis (CCA) implemented in Seurat to align samples. The aligned CCs were then used for downstream dimensionality reduction and clustering analyses. Total cell clustering was performed by "FindClusters" function at a resolution of 2.0 and the first 30 CCs were used to define cell identity. Dimensionality reduction was performed with "RunUMAP" function and visualized by Uniform Manifold Approximation and Projection (UMAP). For subgroup cell clustering, cells of different types were extracted separately and clustered by their respective first 10 CCs using resolutions of 0.5 for mitotic cells and 0.8 for fibroblasts. Epidermal cells were clustered by the first 21 CCs using a resolution of 1.8. Marker genes for each cluster were determined with the Wilcoxon rank-sum test by "FindAllMarkers" function. Only those with |'avg_logFC'| > 0.25 and 'p_val_adj' < 0.05 were considered as marker genes.

Functional enrichment analysis

For gene ontology (GO) enrichment analysis, we obtained differential expressed genes (DEGs) for each cell subset by setting log2foldchange as 0.4 and observed in at least 30% of cells via FindAllMarkers function wrapped in Seurat package. Then, enrichment analysis was performed with DEGs using clusterProfiler packages.

Pseudotime analysis

R package Monocle2 [3] (version 2.99.3) was used to reconstruct the epidermal cell developmental trajectory. The UMI matrix was used as input and variable genes obtained from epidermal cell types were detected by Seurat to sort cells in pseudotime. 'UMAP' was applied to reduce dimensions and the visualization functions "plot_cell_trajectory" were

used to plot each group along the same pseudotime trajectory.

Regulatory analysis of transcription factors

To infer transcription factor (TF)–target interactions, single-cell regulatory network inference and clustering (SCENIC) algorithm was run with raw expression matrices to identify regulons specifically involving different cell subsets. TF activities (AUCell) for each cell were calculated with motif collections version mc9nr. The significantly upregulated regulon was defined as log fold change of more than 0.1 and adjusted p-value < 10−5.

In this study, we retrieved targeted genes that were positively correlated with selected TF) from SCENIC output “regulon” file for network analysis visualization. The transcriptional network of TF and predicted target genes were visualized by Complexheatmap package and Cytoscape (v3.8, Seattle, WA, USA).

Additionally, we performed coexpression analysis to further support abovementioned gene expression network analysis. In brief, the log-transformed normalized expression matrix was extracted from cell subset of interest. Then, Pearson correlation was calculated for all genes, and only genes with correlation greater than 0.15 and p value lower than 0.05 were kept.

Cell-cell interaction analysis

CellChat [4] (v 1.1.3) R package was used for cell-to-cell ligand-receptor interaction analysis. Firstly, the normalized expression matrix was imported and the cellchat object was created through the create CellChat function. Use the default parameters through identify overexpressed genes, identify overexpressed Interactions and preprocessing operations projectData function; The computeCommunProb, filter Communication

(min.cells = 10) and computeCommunProbPathway functions were used to calculate potential ligand-receptor interactions. Finally, the intercellular communication network is aggregated by aggregate Net function.

Cell viability assay

HaCaT and WS1 cells were seeded in a 96-well plate at a density of 5×10^3 cells per well, and then cells were treated with C-DIM8 (20 μM). *In vitro* viability was measured using the Cell Counting Kit-8 (CCK-8) (Dojindo Laboratories, Kumamoto, Japan). Optical density was measured at 450 nm using a micro-plate reader (Biotek, Winooski, VT).

Cellular reactive oxygen species (ROS) assay

HaCaT and WS1 cells were seeded in triplicate in 6-well plates, cultured overnight, and treated with DMSO and 20 μM C-DIM8 for 24 h before 0/20 Gy irradiation (10 Gy irradiation for WS1 cells). The levels of ROS in HIEC cells were determined using the ROS sensitive dye 2',7'- dichlorofluorescein diacetate (DCF-DA) (Beyotime, Nantong, China), which is converted by ROS into the highly fluorescent 2' ,7'-dichlorofluorescein (DCF). The cells were incubated with 10 mM DCF-DA for 20 min at 37 ˚C and subsequently washed with phosphate buffer saline (PBS). The cells infected with ad-NC and ad-Nur77 were stained with dihydroethidium (DHE) (Beijing Solarbio Science & Technology Co., Ltd, Beijing, China). The level of ROS was performed using FACS Celesta flow cytometer (BectonDickinson, Franklin Lakes, NJ). FlowJoTM (Version 10.7) was used to analyze the data.

Cell apoptosis assay

Annexin V/propidium iodide (PI) double-staining analysis was used to detect cell apoptosis.

HaCaT and WS1 cells were seeded in triplicate in 6-well plates, cultured overnight and treated with or without C-DIM8 (20 μM) before irradiation. After 48 h, the cells were stained with fluorescein FITC-conjugated Annexin V and PI (Yeason, Shanghai, China), the cells infected with ad-NC and ad-Nur77 were stained with Annexin V-APC/7-AAD Apoptosis Kit (Multi Sciences, Zhejiang China), The apoptosis analysis was performed using FACS Celesta flow cytometer (Becton Dickinson, Franklin Lakes, NJ), FlowJo™ (Version 10.7) was used to analyze data.

Clonogenic assay

For standard clonogenic assays, cells were re-suspended and seeded into six-well plates at 200 cells/well, cells were incubation C-DIM8 than 2, 4, 6 Gy X-rays irradiation. The cells were grown from 7-10 d to allow for colony formation and were subsequently fixed and stained using crystal violet. Colonies consisting of >50 cells were counted as a clone.

Lactate dehydrogenase release assay

To further measure the extent of cellular damage, lactate dehydrogenase (LDH) activity was also tested. After incubation, the culture supernatants were collected. The activity of LDH was detected at 490 nm according to the LDH assay kit (Beyotime). The LDH activity is measured by a 2-step reaction. At the first step, LDH catalyzes the reduction of NAD^+ to NADH coupled with the oxidation of lactate acid to pyruvic acid. At the second step, diaphorase as a coupling enzyme uses NADH to catalyze the reduction of tetrazolium INT to formazan.

Table S1 The sequence of primers used in this study.

Primers	Sequence

Nur77 forward	AGGGCTGCAAGGGCTTCT
Nur77 reverse	GGCAGATGTACTTGGCGTTTTT
GAPDH forward	CAACGGATTIGGTCGTATT
GAPDH reverse	CACAGTCTTCTGGGTGGC

Table S2 The antibodies used in this study.

Antibodies	Source	Identifier (Cat number)
Anti-PARP antibody	Cell Signaling Technology	9532
Anti-cleaved PARP antibody	Cell Signaling Technology	5625
Anti-Bax antibody	Cell Signaling Technology	2722
Anti-Bcl2 antibody	Cell Signaling Technology	3498S
Anti-Caspase 3 antibody	Cell Signaling Technology	9662
Anti-Cleaved Caspase 3 antibody	Cell Signaling Technology	9661
Anti-Caspase-1 antibody	Abcam	ab207802
Anti-Cleaved-Caspase 1 (Asp297) Antibody	Cell Signaling Technology	4199
Anti-Gasdermin D antibody	Cell Signaling Technology	39754
Anti-Cleaved Gasdermin D antibody	Cell Signaling Technology	36425

Anti-RIP antibody	Cell Signaling Technology	3493
Anti-p-Phospho-RIP antibody	Cell Signaling Technology	65746S
Anti-MLKL antibody	Cell Signaling Technology	14993
Anti-Phospho-MLKL antibody	Cell Signaling Technology	91689

References

1. Kourtis N, Wang Q, Wang B, Oswald E, Adler C, Cherravuru S, et al. A single-cell map of dynamic chromatin landscapes of immune cells in renal cell carcinoma. Nat Cancer. 2022; 3(7): 885-98.

2. Butler A, Hoffman P, Smibert P, Papalexi E, Satija R. Integrating single-cell transcriptomic data across different conditions, technologies, and species. Nat Biotechnol. 2018; 36(5): 411-20.

3. Qiu X, Mao Q, Tang Y, Wang L, Chawla R, Pliner HA, Trapnell C. Reversed graph embedding resolves complex single-cell trajectories. Nat Methods. 2017; 14(10): 979-82.

4. Jin S, Guerrero-Juarez CF, Zhang L, Chang I, Ramos R, Kuan CH, et al. Inference and analysis of cell-cell communication using CellChat. Nat Commun. 2021; 12(1): 1088.

Supplementary Results

Figure S1

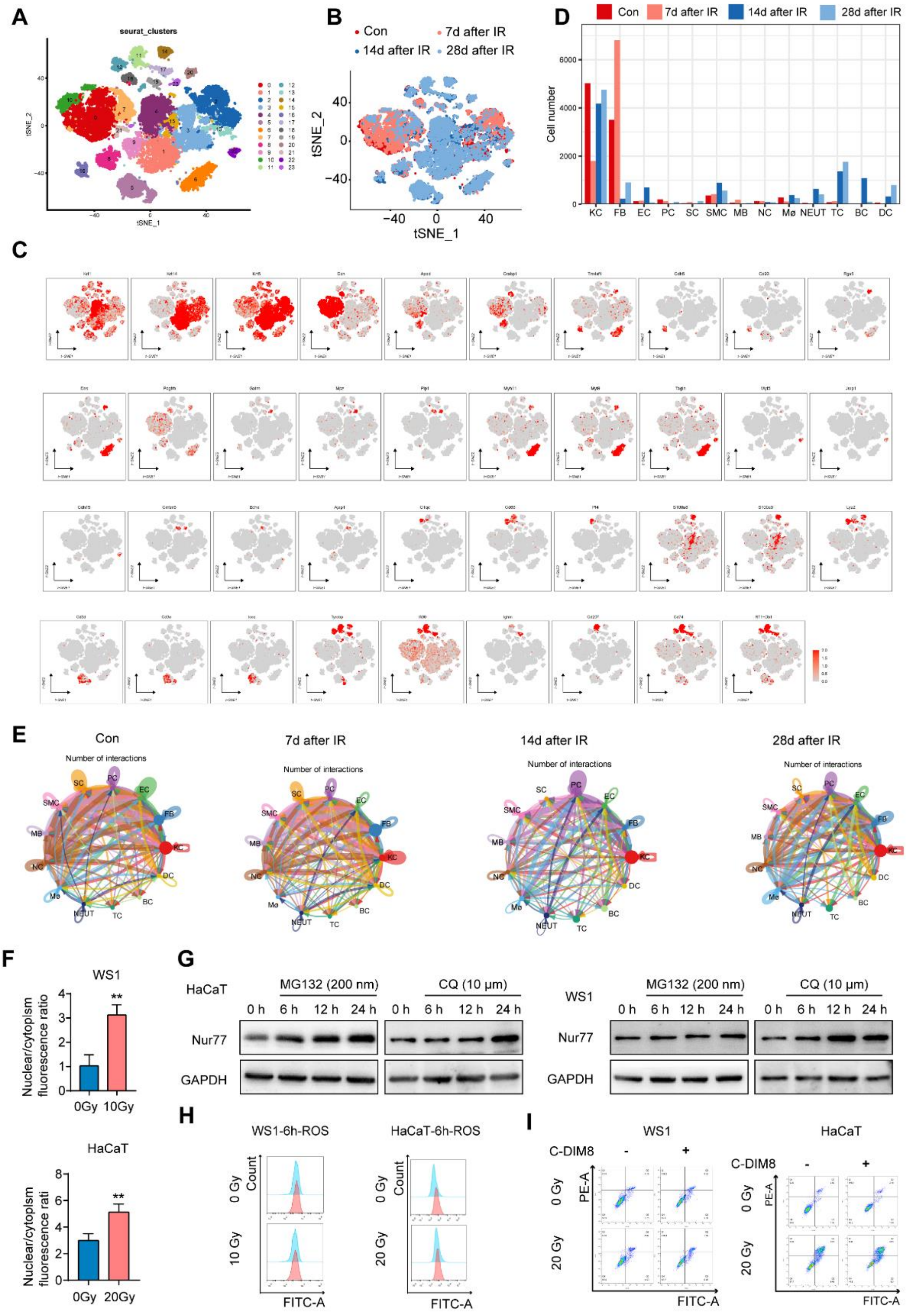

Figure S1. (A) t-SNE plot of 30281 cells from rat skins of control, 7 days after radiation, 14 days after radiation, and 28 days after radiation. (B) t-SNE plots showing the dynamic

changes in all cell clusters in the skin among each group. (C) t-SNE expression plots display cell type-specific marker gene expression. (D) Numbers of all cells belonging to the skin cell types among each group. (E) Circle network plots showing number of cell-cell interactions generated with CellChat in different groups. (F) Statistical chart of nuclear-cytoplasmic ratio of Nur77 fluorescence intensity in immunofluorescence results. (G) The effect of MG132 and CQ on Nur77 expression as determined by Western blotting analysis in WS1 and HaCaT cells. (H) The effect of C-DIM8 on ROS production after different dose of irradiation as determined by DCFH-DA staining in WS1 and HaCaT cells. (I) The effect of C-DIM8 on cell apoptosis determined by AV/PI staining in WS1 and HaCaT cells.
